# Superior Rectal Artery Preservation in Laparoscopically Assisted Subtotal Colectomy and Ileorectal Anastomosis for Slow-Transit Constipation

**DOI:** 10.3390/biomedicines12050965

**Published:** 2024-04-26

**Authors:** Ta-Wei Pu, Yu-Hong Liu, Jung-Cheng Kang, Je-Ming Hu, Chao-Yang Chen

**Affiliations:** 1 Division of Colon and Rectal Surgery, Department of Surgery, Songshan Branch, Tri-Service General Hospital, National Defense Medical Center, Taipei 10581, Taiwan; tawei0131@gmail.com; 2 Division of Colorectal Surgery, Department of Surgery, Tri-Service General Hospital, National Defense Medical Center, Taipei 11490, Taiwan; lovewithoutreasons@gmail.com (Y.-H.L.); jeminghu@gmail.com (J.-M.H.); 3 Division of Colon and Rectal Surgery, Department of Surgery, Taiwan Adventist Hospital, Taipei 10556, Taiwan; jckang5534@gmail.com

**Keywords:** slow transit constipation, superior rectal artery, anastomosis leakage

## Abstract

Our previous retrospective observational study demonstrated the safety of laparoscopically assisted subtotal colectomy with ileorectal anastomosis and preservation of the superior rectal artery (SRA), without instances of leakage, in patients with slow-transit constipation (STC). Thus, we extended the enrollment period and enlarged the sample size to detect the differences in the postoperative complications and surgical and functional outcomes between patients who underwent laparoscopically assisted subtotal colectomy with and without SRA preservation. We conducted a retrospective single-center analysis of patients with STC who underwent laparoscopically assisted subtotal colectomy between 2016 and 2020. The diagnosis of STC was based on the colonic transit and anal functional tests and barium enema to exclude secondary causes. Patients were divided into group A, which underwent surgery with SRA preservation, and group B, which underwent ligation of the SRA during surgery. Outcome assessments for both groups included the incidence of anastomotic breakdown, intraoperative complications, length of hospital stay, estimated blood loss, time to first flatus, and complications. Propensity score matching allocated 34 patients to groups A and B each. Postoperative bowel function, including time to first flatus, stool, and oral intake, recovered better in group A than in group B. Anastomotic leakage, a significant postoperative complication, was less frequent in patients with SRA preservation. In conclusion, preservation of the SRA in patients undergoing laparoscopically assisted subtotal colectomy with ileorectal anastomosis for STC is associated with favorable postoperative bowel function recovery and lower anastomotic leakage rates.

## 1. Introduction

Constipation is a condition characterized by infrequent bowel movements, difficulty in passing stool, or a feeling of incomplete bowel movements; the causes vary, and one of the mechanisms is slow transit of the colon [1,2].

Although slow-transit constipation (STC) has traditionally been classified as a functional disorder, recent clinical and manometric evidence suggests that most motility alterations in STC may be neuropathic in origin. This indicates that STC may be caused by impairment of the nerves that control the muscles involved in bowel movements, rather than simply being a result of decreased motility of the digestive system. This new under-standing of the mechanisms underlying STC may lead to the formulation of more targeted and effective treatment options [3]. The diagnosis of STC often requires a colonic transit test that uses radiopaque markers to track the movement of fecal matter through the colon. Delay in emptying of these markers can indicate a problem with digestive system motility, which is characteristic of STC [4,5,6]. Surgical intervention with subtotal colectomy may be necessary for STC without pelvic outlet obstruction that does not respond well to conservative treatment with laxatives. This procedure can help improve motility and reduce the symptoms of constipation. However, as with any surgery, the risks and potential complications must be carefully considered before selecting this treatment option. It is important for patients to discuss the risks and benefits of surgery with their healthcare provider to facilitate informed decision making [7]. Anastomotic leakage is a potentially serious and most undesirable complication of colorectal surgery [8]. It is reportedly responsible for a postoperative mortality rate as high as 40%, prolonged hospitalization, and an increase in the overall healthcare costs due to the requirement of sepsis treatment and additional surgical intervention to repair the leak [9]. Therefore, the objective of this study was to investigate the feasibility, utility, and outcomes for patients with STC who underwent superior rectal artery (SRA)-preserved laparoscopically assisted subtotal colectomy.

## 2. Materials and Methods

This study incorporated a retrospective single-center design. The decision to preserve the SRA during laparoscopically assisted subtotal colectomy for STC was left to the surgeon’s discretion. Patients who were treated at the Division of Colon and Rectal Surgery at the Taiwan Adventist Hospital and diagnosed with constipation (according to Rome II criteria) between January 2016 and January 2020 were evaluated using laboratory tests including thyroid function tests, serum calcium, serum glucose, and complete blood counts. All patients underwent clinical evaluation, including digital rectal examination and psychological consultation. Patients with other conditions, such as colonic obstruction and drug-induced constipation, were excluded. The diagnosis of STC was based on a series of diagnostic tests, including the colonic transit test, anorectal manometry, balloon expulsion test, and barium enema. A positive colonic transit test was defined as radiopaque marker stasis in the colorectum exceeding 20% 120 h after swallowing all of them. Anorectal manometry and balloon expulsion tests were performed to ensure that there was no outlet-obstructed defecation and to rule out pelvic floor dysfunction. This thorough diagnostic work-up facilitated accurate diagnosis of STC and the exclusion of other potential causes of constipation, which helped in the implementation of more effective and targeted treatment approaches. Patients underwent a barium enema to ensure that there were no mechanical obstruction problems, and all cases showed a redundant colon. Colonoscopy findings were normal in all patients, and anal ultrasonography did not reveal disruption of the external anal sphincter. Patients with a colonic transit test time greater than 96 h but whose barium enema, balloon expulsion test, anal manometry, colonoscopy, and anal ultrasonography results were normal were deemed suitable for inclusion in this analysis. Informed consent was obtained from all patients prior to examination. Since January 2016, the surgical team has attempted to preserve the SRA in every patient with STC. All surgeries were performed by the same team. This consistent approach helped ensure uniformity and minimized variations in surgical techniques or patient selection, which could affect the study results.

We enrolled 100 patients diagnosed with STC who underwent laparoscopically assisted subtotal colectomy between January 2016 and January 2020. Data on various factors were recorded, including age, body mass index, preoperative laxative dependence, preoperative defecation duration, colonic transit time, operative time, largest incision length, volume of blood loss, operative complications, postoperative bowel movements, length of hospital stay, and functional outcomes. These data were used to analyze the feasibility, utility, and outcomes for patients with STC who underwent SRA-preserved laparoscopically assisted subtotal colectomy.

This study was conducted in accordance with the Declaration of Helsinki and ap-proved by the Institutional Review Board of Taiwan Adventist Hospital (TAHIRB No. 105-E-10). Approval from the institutional review board indicates that our study protocol has undergone thorough ethical review and that the institutional review board has determined that waiving informed consent is justified. All the personal information from the participant in this study was deidentified prior to analysis.

### 2.1. Statistical Analysis

This study collected data from 100 patients diagnosed with slow-transit constipation (STC) between January 2016 and January 2020. These patients underwent laparoscopically assisted subtotal colectomy with ileorectal anastomosis (IRA), and they were divided into two groups based on the preservation or sacrifice of the SRA, with 59 patients in the preservation group (group A) and 41 in the sacrifice group (group B).

Patient characteristics were presented using percentages and mean ± standard deviation. Propensity score matching (PSM) analysis was performed by matching age, sex, and body mass index, while establishing a tolerance level of 0.05 to reduce the effects of confounding variables.

During the data processing phase, rigorous statistical analyses were conducted to ensure the accuracy and reliability of the results. Comparisons between group A and group B were primarily conducted using independent-sample *t*-tests, which are suitable for assessing differences in mean values between two groups, such as surgical time, hospital stay, and the time to first flatus and oral intake. Additionally, Chi-square tests were used to analyze categorical variables, such as the incidence of postoperative complications. Where applicable, other statistical methods, including but not limited to paired-sample *t*-tests and Analysis of Variance (ANOVA), were also employed to further validate our research hypotheses.

All statistical analyses were performed using SPSS statistical software (version 22.0, IBM Corp., Armonk, NY, USA). An omnibus test was conducted to ensure the overall appropriateness of the statistical models. In all statistical tests, a *p*-value of less than 0.05 was considered statistically significant.

### 2.2. Surgical Technique

Two highly experienced colorectal surgeons, who perform over 100 laparoscopic and open colorectal cancer procedures annually, conducted laparoscopic surgeries entailing preservation of the SRA in patients with STC. The planned procedure for all cases was laparoscopic subtotal colectomy with IRA under general anesthesia, with the patients placed in the modified lithotomy position. The laparoscopic procedure involved the use of five trocars; the primary trocar was a 10 mm port inserted above the umbilicus to establish a pneumoperitoneum for the laparoscope. Four working ports of different sizes were established as follows: a 12 mm port at the right iliac fossa, a 10 mm port at the left iliac fossa, and two 5 mm ports in the right and left upper quadrants of the abdomen. Mobilization of the colon segments was initiated from the right colon using the LigaSure device.

**Figure 1:**
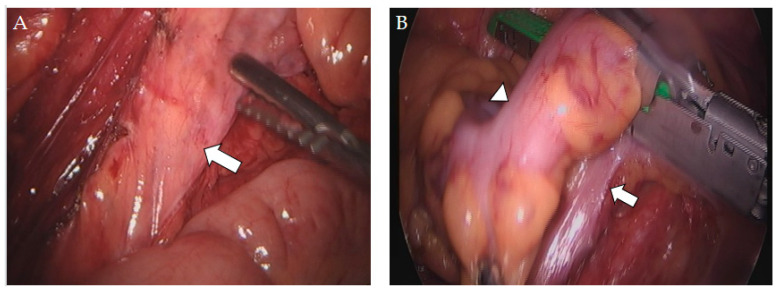
Intraoperative images of preserving the superior rectal artery. (**A**) Preservation of the superior rectal artery (arrow) in laparoscopically assisted subtotal colectomy with ileorectal anastomosis. (**B**) Sigmoid–rectal junction (arrowhead) and sparing of the superior rectal artery (arrow).

All patients in group A underwent laparoscopic subtotal colectomy with preservation of the SRA, which was performed by two experienced colorectal surgeons (Figure 1). The surgical procedure involved mobilization of the colon segments, resection of the colon with a laparoscopic linear stapler at the junction of the rectum and the sigmoid colon, and mobilization of the rectal stump for transanal insertion of a circular stapler or Hegar dilator.

A 4–5 cm long surgical incision was made in the Pfannenstiel area, and the mobilized bowel section was brought out. The end of the ileum was separated a few centimeters be-fore the ileocecal valve, and the anvil of a circular stapling device was inserted into its lumen. The anvil-secured ileum was placed back into the abdominal cavity, and a circular stapling device was used to perform a transanal end-to-end anastomosis. A Jackson-Pratt drain was placed in the pelvis, followed by layer-by-layer wound closure.

## 3. Results

A total of 100 patients diagnosed with STC underwent laparoscopically assisted subtotal colectomy with IRA between January 2016 and January 2020. Table 1 displays the participants’ preoperative characteristics. The SRA was preserved in 59 patients (group A) and sacrificed in 41 patients (group B). All patients had been diagnosed with STC prior to the procedure, and each patient had severe constipation with an average defecation duration of 7.9 and 9.3 days, respectively. There were 74.6% females in group A and 80.5% females in group B. Before surgery, almost all patients reported intermittent abdominal pain (55 and 36 patients in groups A and B, respectively). Except for the colonic transit time, which was 148.2 and 141 h in groups A and B, respectively, the preoperative characteristics did not differ significantly between the two groups.

The PSM analysis was performed with age-, sex-, and body-mass-index-matched patients, with 34 patients allocated to each group (Table 2). The significant difference in the preoperative colonic transit time between the two groups (148.4 and 138.2 h) was retained even after the PSM analysis, but shorter time to first flatus was noted in the group with a longer colonic transit time before the surgery.

Table 3 outlines the intra- and postoperative characteristics of the patients. The surgical time with preservation of the SRA was 142.8 ± 28.8 min, while surgery with ligation required 148.7 ± 33.6 min (*p* = 0.439). The average length of hospitalization did not differ significantly between group A (9.9 ± 3.2 days) and group B (10.1 ± 2.6 days) (*p* = 0.806). However, the average time of first flatus was significantly shorter in group A (2.8 ± 0.9 days) than in group B (4.2 ± 1.5 days) (*p* < 0.001). Similarly, the average time of oral intake was significantly shorter in group A (2.9 ± 0.8 days) than in group B (4.3 ± 1.1 days) (*p* < 0.001).

No intraoperative complications were observed (Table 4). Anastomotic breakdown did not occur in group A, whereas it occurred in four patients (11.8%) in group B (*p* = 0.039). The incidence of postoperative ileus or urinary tract infections did not differ significantly between the two groups. Postoperative complications, such as incisional hernia, developed in only one patient (1.5%) in group A, and no wound infections were observed in either group. The Jackson-Pratt drain was removed on the day of discharge, and there were no surgery-related mortalities. No patient required conversion to exploratory laparotomy.

## 4. Discussion

Currently, total colectomy followed by IRA is considered the standard of care for patients with STC who are refractory to conservative treatment [10]. There are different ways to perform the surgery, and we had started minimally invasive total colectomy for STC patients with a hand-assisted model, which showed good results [11]. Currently, we perform laparoscopic surgery instead, and more and more precise techniques could be adopted. Ischemia of the anastomotic region is one of the most important factors leading to anastomotic leakage. It is intuitive to preserve the SRA while performing total colectomy with IRA for STC patients, and we have mostly focused on anatomy for non-cancerous diseases. Different from the disease in our study, there are some concerns related to performing surgery for malignancies while preserving the SRA for sigmoid or upper rectum lesions [12]. 

We performed a case-control study on the basis of the results of our previous single-center, observational study that included 32 patients [13]. The cross-matched case-control study enrolled 34 patients with SRA preservation who were in the preservation group. Many of the studies analyzed in a systematic review found that constipation is more prevalent among females, which is also noted in our study. The higher prevalence of constipation among females is thought to be influenced by hormonal factors. For instance, women may experience a greater risk of constipation during the luteal phase of their menstrual cycle due to hormonal changes. Additionally, progesterone, particularly during pregnancy, can contribute to constipation. Furthermore, women may experience damage to their pelvic floor muscles during childbirth or gynecological surgery, which can also contribute to constipation. The incidence of anastomotic leakage after gastrointestinal surgery varies according to the localization of the anastomosis (all resections: 4.3–13%) [14]. Given the devastating consequences of anastomotic leakage in colorectal surgery, numerous studies have explored the impact of rectal blood supply on anastomotic healing, with special emphasis on the preservation of the inferior mesenteric artery. However, research on the effects of SRA on anastomotic healing and postoperative complications is limited, particularly in patients with STC. In addition to reducing the risk of anastomotic leakage, the SRA-sparing technique may also preserve the hypogastric nerve plexus or inferior mesenteric plexus, which can potentially improve functional outcomes [15].

Bergamaschi et al. investigated 30 patients who underwent laparoscopic SRA-preserving sigmoidectomy for complete rectal prolapse and found no anastomotic leakage [16]. Tocchi et al. reported a similar finding in their randomized controlled trial, where they observed a significantly lower rate of anastomotic leakage in the group of patients undergoing left colectomy for diverticular disease while preserving the SRA [17]. Sohn et al. have also demonstrated that preserving the SRA may be associated with a reduced rate of anastomotic leakage in patients undergoing laparoscopic sigmoid resection for diverticular disease [15]. In our study, no anastomotic leakage and faster postoperative bowel function recovery were noted in the SRA-preserving group. 

The application of laparoscopic techniques to colon surgery has been successful in reducing morbidity, mortality, and length of hospital stay [18,19]. The length of hospital stay typically ranges from 7 to 13 days in patients with STC [20,21,22]. However, postoperative ileus can complicate the treatment course for many patients following surgery [7]. Although we did not find a lower postoperative ileus rate in our study, earlier first flatus after the operation in the SRA-reserving group enabled early oral intake after surgery.

The key limitations of our study were its retrospective design and small sample size. Many new devices show up nowadays, like indocyanine green (ICG) fluorescence imaging, and the new techniques provide more objective evaluations. This could test our SRA preservation surgery and lower the risk for those with concerns of ischemia upon anastomosis in other methods. Unfortunately, our hospital did not start ICG fluorescence until 2022, and further research on the effect of the ICG test between two group is being conducted in our hospital. Despite these limitations, we report a pilot study comparing the benefits of SRA preservation in subtotal colectomy with ileorectal anastomosis for STC.

## 5. Conclusions

Preservation of the SRA in patients with STC undergoing laparoscopically assisted subtotal colectomy with ileorectal anastomosis was associated with more favorable postoperative bowel function and a lower risk of anastomotic leakage with better recovery during hospital course compared with that without the preservation.

## Figures and Tables

**Table 1:** Preoperative variables before propensity score matching.

Variables	Group A (*n* = 59)	Group B (*n* = 41)	*p*-Value
Age (years)	39.4 (28.7~50.1)	42.9 (31.9~43.9)	0.120
Body mass index (kg/m^2^)	24.6 (20.9~28.3)	25.8 (22.7~28.9)	0.100
Female (%)	44 (74.6)	33 (80.5)	0.490
Preoperative laxative-dependent (years)	13.9 (8.0~19.8)	14.1 (8.3~19.9)	0.846
Preoperative defecation duration (days)	7.9 (4.2~11.6)	9.3 (5.4~13.2)	0.083
Colonic transit time (h)	148.2 (131.4~165.0)	141 (119.4~162.6)	0.065
Previous abdominal surgery	13 (22)	5 (12.2)	0.208
Preoperative abdominal pain	55 (93.2)	36 (87.8)	0.352

Values with mean (±SD) or total number (percentage).

**Table 2:** Preoperative variables after propensity score matching.

Variables	Group A (*n* = 34)	Group B (*n* = 34)	*p*-Value
Age (years)	42.3 (31.3~53.3)	41.7 (30.6~52.8)	0.827
Body mass index (kg/m^2^)	25.8 (22.1~29.5)	25.9 (22.7~29.1)	0.902
Female (%)	24 (70.6)	28 (82.4)	0.253
Preoperative laxative-dependent (years)	14.3 (8.7~19.9)	14.2 (9.0~19.4)	0.929
Preoperative defecation duration (days)	8.0 (4.4~11.6)	9.5 (5.7~13.3)	0.100
Colonic transit time (h)	148.4 (131.0~165.8)	138.2 (118.7~157.7)	0.025
Previous abdominal surgery	7 (20.6)	2 (5.9)	0.074
Preoperative abdominal pain	32 (94.1)	30 (88.2)	0.393

Values with mean (±SD) or total number (percentage).

**Table 3:** Surgical and postoperative variables.

Variables	Group A (*n* = 34)	Group B (*n* = 34)	*p*-Value
Operative time (min)	142.8 (114.0~171.6)	148.7 (115.1~182.3)	0.439
Estimated blood loss (mL)	104.3 (56.2~150.4)	133.5 (90.2~176.8)	0.009
Time to first flatus (days)	2.8 (1.9~3.7)	4.2 (2.7~5.7)	<0.001
Time to first stool passage (days)	2.7 (2.1~3.3)	2.4 (1.9~2.9)	0.037
Time to oral intake (days)	2.9 (2.1~3.7)	4.3 (3.2~5.4)	<0.001
Dose of Demerol administered (mg)	100 (65~135)	110 (70~150)	0.359
Duration of hospital stay (days)	9.9 (6.7~13.1)	10.1 (7.5~12.7)	0.806
Postoperative bowel frequency (per day)	2.2 (1.2~3.2)	2.3 (1.3~3.3)	0.597

Values with mean (±SD) or total number (percentage).

**Table 4:** Intraoperative and postoperative complications.

Characteristics	Group A (*n* = 34)	Group B (*n* = 34)	*p*-Value
Intraoperative complications			
None	34 (100%)	34 (100%)	
Postoperative complications			
Urinary tract infection	3 (8.8%)	3 (8.8%)	1
Ileus (over 5 days)	4 (11.8%)	2 (5.9%)	0.393
Incisional hernia	1 (1.5%)	0 (0%)	0.314
Wound infection	0 (0%)	0 (0%)	1
Anastomosis leakage	0 (0%)	4 (11.8%)	0.039

Values with mean (SD) or total number (percentage).

## Data Availability

No new data were created or analyzed in this study. Data sharing is not applicable to this article.
